# Barriers to Diet and Exercise among Nepalese Type 2 Diabetic Patients

**DOI:** 10.1155/2017/1273084

**Published:** 2017-11-14

**Authors:** Saruna Ghimire

**Affiliations:** ^1^Valley College of Technical Sciences, Kathmandu, Nepal; ^2^Agrata Health and Education Development (AHEAD)-Nepal, Kathmandu, Nepal

## Abstract

This study aims to identify the modifiable barriers encountered by type 2 diabetic patients in Nepal to achieving their recommended dietary and exercise advice. A cross-sectional study was conducted among 197 type 2 diabetic patients, attending a diabetic clinic. Binary logistic regression models were used to identify perceived barriers. About 41% and 46% of the participants were noncompliant to diet and exercise advice, respectively; only 35.5% the participants were compliant to both. Perceived social acceptability (OR = 0.14; 95% CI: 0.03–0.58) and reminder to action (OR = 2.77; 95% CI: 1.38–5.53) were associated with noncompliance to diet. Most of the barriers to diet were related to taste, feast and festivals, lack of knowledge, and availability of healthy options. Self-efficacy (OR = 0.09; 95% CI: 0.02–0.34) and social acceptability (OR = 0.12; 95% CI: 0.04–0.34) were significant predictors of noncompliance to exercise. The supportive role of children and spouse and the opposing role of friends and relatives were important for compliance to both. A misconception on diabetes severity, effectiveness of healthy lifestyle, and exercise timing was prevalent among the study participants. Addressing the modifiable barriers identified in this study is essential for successful diabetes management in Nepal.

## 1. Background

In Nepal, type 2 diabetes mellitus (T2DM) is the third most common noncommunicable disease among hospitalized patients [1], with an estimated prevalence of 6.3%–25.9% [1–3] among the general population. Unhealthy diet and physical inactivity are important modifiable risk factors for T2DM [4]. Adoption of a healthier lifestyle is a key factor in prevention [4] and management [5] of T2DM. Effective lifestyle interventions that include healthy diet and exercise can reduce diabetes incidence up to 55% [6] and have shown to be more efficient than antidiabetic medicines [7]. Additionally, diet combined with physical exercise has been identified as the most effective preventative strategy in reducing the incidence of diabetes [8]. Despite their importance, the practice of both is relatively low among Nepalese diabetic patients. In a previous study, two-thirds of diabetic patients were advised by a health professional to eat a special diet and to start or do more exercise [3]. However, none of the patients was adherent (87.5% nonadherence and 12.5% poor adherence) to dietary advice, and only 21% were adherent to exercise [9].

A previous study from Nepal identified demographic factors associated with nonadherence to dietary advice [9]. Similarly, negative family history of diabetes, divorced status, and lower socioeconomic class determined nonadherence to physical activity [9]. However, literature is lacking to explore the modifiable psychosocial factors that prevent people from adherence. Nepal has a unique sociocultural context. Therefore, inferences on psychosocial barriers to health behaviors cannot be made from studies conducted in different contexts.

To motivate people to adopt a healthy lifestyle, a solid understanding of the barriers, especially those modifiable, encountered by those under diet and exercise medical advice is necessary. Health professionals can better facilitate behavior change by identifying such barriers to compliance [10]. Hence, this study aims to identify the barriers encountered by T2DM patients in Nepal to achieving their recommended dietary and exercise changes as advised.

## 2. Methods

### 2.1. Study Design and Setting

A cross-sectional study was conducted in March–May, 2016, among T2DM patients at the outpatient department (OPD) of the Diabetes, Thyroid & Endocrinology Care Centre (DTECC) in Kupondole, Nepal. DTECC-Nepal was chosen because it is a specialized diabetic center offering clinical and educational services for diabetes, thyroid, and other endocrine-related disorders.

### 2.2. Ethics Approval and Consent to Participate

The Ethical Review Board of the Nepal Health Research Council granted ethical approval for this study. Permission was also obtained from the DTECC-Nepal. Informed written consent was taken from each respondent, participating voluntarily. The identity of participants was kept confidential.

### 2.3. Participants

*Eligible Participants*. (i) They were at least 18 years old; (ii) diagnosed with T2DM; (iii) had received advice from their consultants to follow a special diet appropriate for diabetes patients and to perform exercises; and (iv) had an OPD card (local medical record) that reflected their disease status and previous consultations. Patient's diabetic status and other eligibility criteria were confirmed by their OPD card. Patients with thyroid, endocrine, and other severe systemic comorbidities that could limit their dietary options and exercise activity were excluded. Pregnant women and physically disabled patients were excluded.

### 2.4. Study Size and Sampling

The sample size of 197 individuals was calculated using StatCalc in Epi Info 7 [11] with 5% alpha or Type I error, 5% margin of error, and 14.6% prevalence of diabetes in urban Nepal [12]. Systematic random sampling was used for selecting samples. Data was collected from every third eligible patient from OPD list until the desired sample size was achieved.

### 2.5. Data Collection

Individual interviews were conducted during the patient waiting time. Surveyors had baccalaureate in public health and were provided with a one-day extensive orientation on the tool, sampling strategy, and data collection techniques. The study tool was pretested among 20 patients meeting the study inclusion criteria at a different diabetes center, Kathmandu Diabetes & Thyroid Centre (Alka Hospital) in Lalitpur, Nepal. Pretest responses were not included in the final analysis.

### 2.6. Variables

#### 2.6.1. Outcome Variables

This study has two binary outcome variables: compliance with dietary advice and compliance with exercise advice. Since diet recommended to a diabetic patient is personalized, the food regimen recommended by the consultants during diabetic care consultation was considered as a healthy diet/option for that patient. Therefore, compliance to diet (dichotomized, yes/no) was defined following the recommended dietary advice at least six days a week. Otherwise, they were classified as noncompliant to diet. Hereafter, healthy diet refers to the dietary regimen advised by the consultants to the patients. Since there are no national guidelines on physical activity for the Nepalese, we defined physically active status based on international guidelines [13]. Therefore, compliance to exercise (dichotomized, yes/no) was defined as anyone reporting 5 or more sessions of moderate or vigorous activity per week; otherwise, they were classified as noncompliant to exercise.

#### 2.6.2. Independent Variables

Based on the tenets of the health belief model (HBM) [14] and the theory of planned behavior (TPB) [15], the following seven key determinants of human behavior were examined as a dichotomized (yes/no) response.

*Perceived Self-Efficacy*. Self-efficacy refers to the belief in one's capacity to do a given behavior [16]. Self-efficacy is important for behavior change because if a person believes that he cannot change the behavior, then he will not even attempt to change it [16]. In our study, self-efficacy indicated the respondent's confidence in being able to adhere to the diet and exercise advice. Further, in an open-ended question, we also asked about what made adherence easy and/or difficult.

*Perceived Social Acceptability*. Subjective norms, or perceived social acceptability, evaluates the respondents' perceptions of their significant others' attitudes toward the targeted behavioral change [15]. We asked respondents if most people around them approved/disapproved of their compliance to diet and exercise advice and followed up with open-ended questions about the important people approving or disapproving the compliance.

*Perceived Action Efficacy*. Perceived action efficacy is the perception that the behavior is useful in decreasing the risk of disease or its consequences; people will more likely adopt a behavior when they think that the behavior is beneficial [14, 17]. In our study, we asked participants if the healthy diet (or exercise) was effective in controlling their blood glucose level.

*Cues for Action*. Cues for action determine whether or not a person can remember to do the recommended advice. If someone cannot remember the recommendations that were advised, then other determinants are meaningless. To assess this, we asked the participants how difficult it is to remember to follow the doctor's advice concerning healthy diet or exercise.

*Accessibility of Materials*. We asked the participants how easy it is to get healthy food options and exercising materials.

*Perceived Susceptibility and Perceived Severity*. According to HBM, perceived susceptibility and perceived severity, together known as perceived threat, are the driving factors for behavior change [17]. Perceived susceptibility, or risk, indicates the respondent's perception of his/her likelihood of experiencing diabetes-related complications. Greater perceived risk of disease or its consequences are associated with greater likelihood of adopting the behavior [14].

In addition to these key determinants, we asked several additional questions related to established barriers to dietary compliance (Table 3) to participants noncompliant to dietary advice and administered the barriers to being active quiz (BBAQ) [18] to those noncompliant to exercise advice. The BBAQ is a 21-item instrument (each item measured on a 4-point Likert scale ranging from 0 = “very unlikely” to 3 = “very likely”) that measures barriers to physical activity in seven self-reported constructs: lack of time, social influences, lack of energy, lack of willpower, fear of injury, lack of skill, and lack of resources (Table 5). Internal consistency of BBAQ for our study was 0.64. Approximately 43% of our participants were retired or housewives. Therefore, the response on items related to the availability of time, exercise facilities, and showers at work was very low. A score at or above the 75th percentile of the given subscale was considered an important barrier to exercise.

*Sociodemographic and Lifestyle Variables*. Data on sociodemographic and lifestyle variables, collected by self-report, included age, sex, ethnicity, educational status, occupation, marital status, family type and size, family income, residence, antidiabetic medication, smoking, and alcohol consumption.

### 2.7. Statistical Methods

Data were managed in Epi-Data version 3.1. All analyses were carried out separately for diet and exercise barriers. The demographic characteristics between those who were compliant and those who were noncompliant to diet and exercise were compared by using either Chi-square tests or independent *t*-tests, as applicable. Shapiro-Wilk tests were used to test the normality of quantitative variables. A binary logistic regression model was used to calculate crude and adjusted odds ratios (ORs). Because some of the independent variables showed rare event phenomenon, we used the Firth approach to logistic regression [19]. The multivariate models were adjusted for age, gender, education, smoking, and alcohol. Frequencies of open-ended responses are reported. Data analyses were performed in Stata 13.0 (Stata Corporation, College Station, TX, USA). Two-tailed *P* values less than 0.05 were considered statistically significant.

## 3. Results

### 3.1. Demographic Characteristics of the Study Participants

The study included 197 T2DM patients, 111 males, and 86 females, with a mean age of 54.7 years. Descriptive characteristics of the participants are provided in Table 1. About 41% of the participants were noncompliant to the dietary recommendation, and 46% were noncompliant to exercise recommendation. Only 35.5% of the participants were compliant to both. There was no statistically significant difference, in sociodemographic and behavioral characteristics, between those who were compliant and noncompliant to exercise advice. Similarly, except for smoking and alcohol consumption, there was no difference between those compliant and noncompliant to dietary advice (Table 1).

### 3.2. Barriers to Dietary Recommendation among Study Participants

The majority (98%) of the participants believed that they had sufficient ability, or self-efficacy, to adhere to their dietary advice and doing so was approved by the people in their surroundings (93.4%) (Table 2). About one-fifth of the participants believed that adhering to healthy diet advice will help to control their blood glucose level. A quarter of the participants found it tough to remember to follow their dietary advice. Most of the participants (86%) said that healthy diet is not readily accessible. About 24% of the participants did not believe that diabetes is a serious disease and 34% of the participants did not believe that nonadherence to a healthy diet will put them at risk of diabetic complications. When the noncompliant participants were compared to those compliant to dietary advice, they were found to have lower odds of perceived social acceptability (OR = 0.14; 95% CI: 0.03–0.58) and higher odds of difficulty in remembering the advice (OR = 2.77; 95% CI: 1.38–5.53) (Table 2); findings were statistically significant in both unadjusted and adjusted models.

**Figure 1:**
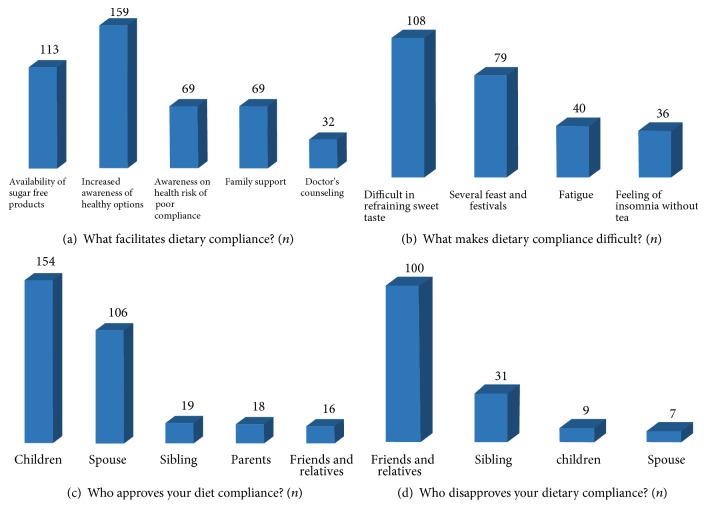
Factors affecting dietary compliance among respondents. Factors that make dietary compliance easier (a) and difficult (b); people approving (c) and disapproving (d) of participant's dietary compliance. All responses are frequencies.

Other aspects of dietary barriers were explored through open-ended follow-up questions (Figure 1). Most of the participants listed increased awareness of healthy options (*n* = 159), availability of sugar-free options (*n* = 133), family support (*n* = 69), and awareness of health consequences of poor compliance (*n* = 69) as factors that may make compliance easier (Figure 1(a)). Difficulty in refraining from sweet tastes (*n* = 108) and several feast and festivals (*n* = 79) made compliance difficult (Figure 1(b)). When asked about people who approved or disapproved of the behavior, the supportive role of children, especially sons (*n* = 145), and spouses (*n* = 106) (Figure 1(c)) was identified as important, as well as the opposing role of friends and relatives (*n* = 100) (Figure 1(d)).

To explore the barriers to diet compliance further, noncompliant participants were asked to express their concordance on various statements that may have resulted in noncompliance (Table 3). Most of the participants who were noncompliant to diet agreed that they lacked the knowledge (79%) and skills to cook/choose healthy options (63%). The majority of them disagreed that their work (58%) and family commitments (72%) keeps them too busy to buy and/or cook healthy food options. A high proportion of noncompliant participants stated that they do not prefer to eat recommended diet (74%); healthy diets are expensive (43%) and unaffordable (58%) (Table 3).

### 3.3. Barriers to Exercise among Study Participants

Taking a morning walk (46.7%, *n* = 92), yoga (15.7%, *n* = 31), and jogging (5.6%, *n* = 11) were the most popular types of exercise among the participants. A quarter (25.9%, *n* = 51) of the participants reported never doing any exercise.

Most of the participants (90%) believed that they are capable of complying with exercise advice (Table 4). About 15% of total participants believed that their significant others would oppose their behavior. More than a quarter of the respondents did not believe that regular exercise was (28%) effective in controlling blood glucose level and that a physically inactive life will lead to severe diabetes complications (30%). Around one-fifth of the respondents did not believe that diabetes was a serious health problem (23%). Difficulty in remembering to exercises was reported by a fifth of the participants, and around 30% said that they had no access to materials and services needed for being physically active. When participants noncompliant to exercise advice were compared to compliant ones, self-efficacy (OR = 0.09; 95% CI: 0.02–0.34) and social acceptability (OR = 0.12; 95% CI: 0.04–0.34) were significant predictors of noncompliance in both unadjusted and adjusted models (Table 4).

**Figure 2:**
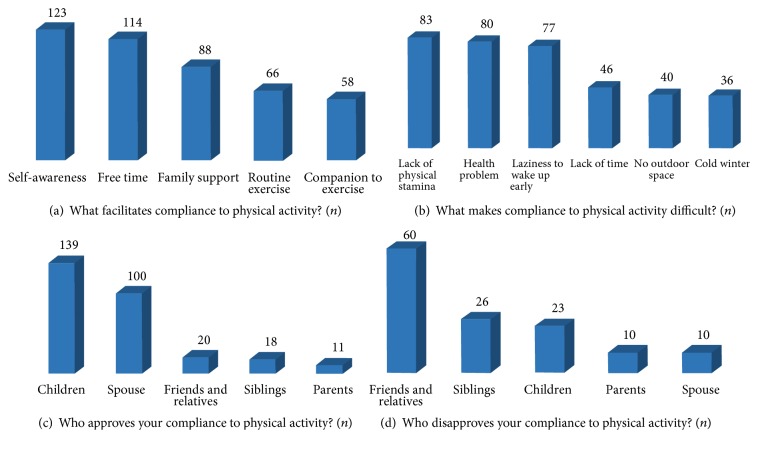
Factors affecting compliance to exercise among respondents. Factors that make compliance to exercise easier (a) and difficult (b); people approving (c) and disapproving (d) of participant's exercise compliance. All response are frequencies.

Open-ended follow-up questions (Figure 2) revealed additional barriers to exercise. Factors such as self-awareness (*n* = 123), having free time (*n* = 114), and family support (*n* = 88) were identified as enabling factors for compliance (Figure 2(a)), whereas, lack of physical stamina (*n* = 83), personal health issue (*n* = 80), and laziness to wake up early in the morning for a morning walk (*n* = 77) were barriers to being physically active (Figure 2(b)). Compliance to exercise advice was supported by children (*n* = 139) and spouses (*n* = 100) (Figure 2(c)), whereas it was opposed by friends and relatives (*n* = 60) (Figure 2(d)).

On barriers to being active quiz (Table 5), participants noncompliant to exercise agreed that lack of energy (68.9%) and lack of willpower (76.7%) were barriers to being physically active. Lack of time (35.6%), fear of injury (36.7), lack of skill (34.4%), and lack of resources (36.7%) were also identified as barriers to being active, but by fewer participants (Table 5).

## 4. Discussion

Despite being advised by the consultant to follow a special diet and perform regular exercise, noncompliance to both was high among our study participants. Healthy lifestyle change can be achieved by the use of behavior change strategies [20] but, to develop such strategies, a clear understanding of underlying barriers is essential. In our study, lack of social acceptability was the main barrier to both diet and exercise among study participants, whereas low perceived self-efficacy was a barrier for exercise and difficulty in remembering was an important barrier to diet among diabetic patients in Nepal.

For both diet and exercise, social acceptability determined compliance. Children and spouses played a supportive role, whereas friends and relatives had an opposing role. Social acceptability was also a facilitator to adherence to dietary [21, 22] and exercise regimens [23, 24] among diabetic patients in other international studies. Additionally, social support was an important facilitator for exercise even among those who lacked self-motivation to exercise [24]. Patients' efforts to maintain and adhere to lifestyle modifications often take place in social settings and thus can alter family and social dynamics [25]. It is believed that social support promotes compliance by encouraging optimism and self-esteem, providing practical help in everyday activities, buffering the stress of living with illness, and reducing patient depression [26]. Interventions that included family support have shown promising results in increasing adherence among diabetic patients who had difficulty adhering to dietary restrictions [27].

Taste and several feast and festivals were important barriers to dietary adherence among study participants; the findings were not unexpected. Barriers to sweet taste can be overcome by using sugar alternatives, that is, natural and artificial sweeteners. Nepal observes several festivals throughout the year and foods are typically rich in sugar, ghee, and a variety of dairy and fats. Food plays a significant role in social events in South Asian traditions, and there is considerable social pressure to eat, especially during festivals [21], which makes compliance to diet difficult.

Study participants also expressed that increased awareness of healthy options would facilitate dietary adherence. Lack of dietary knowledge is associated with poor adherence to diet among diabetic patients [28] because such knowledge makes patients more competent in making informed decisions [29] and enhances self-regulatory capacity to address diet barriers [30]. Such barrier can be addressed both by nutritional counseling of the patient during diabetic care and referral to online resources to those patients who have access to the Internet.

Self-efficacy was a significant determinant of compliance to exercise. Perceived self-efficacy has been associated with physical activity, in general [23] and among diabetes patients [31, 32]. Additionally, interventions incorporating a self-efficacy component were successful in bringing the desired change in physical activity behavior [33]. Individuals with higher perceived self-efficacy are likely to have elevated energy, confidence, and the ability to initiate and maintain physical activity behavior [34]. The stronger the belief one has in their ability, the more likely they will initiate and maintain a regular physical activity in their schedule [16]. The initial stage of adopting a behavior is crucial to increase self-efficacy because success in the adopting phase will increase self-efficacy whereas failure may increase disappointment among individuals [16]. Therefore, individuals who are advised to make small, achievable, and realistic behavioral plans can build up self-confidence and are more likely to be effective at changing behavior patterns [35]. Also, motivational counseling by using role models, such as the compliant participants in our study, may also help to increase self-efficacy among the participants.

One of the important barriers to exercise was laziness to wake up early in the morning for a morning walk. This finding shows a misconception about the timing of exercise among the participants. Also, a large proportion of participants believed that diabetes is not a serious health problem and noncompliance to diet and exercise would not lead to serious diabetes complications. These show a gap in knowledge, which is important to address during diabetic care consultation or through appropriate awareness programs.

Lack of power, energy, or physical stamina and personal health issue were barriers identified in this study as well as in others [36, 37]. Such patients should be encouraged to undertake some form of physical activity despite feelings of tiredness, as ironically such feelings can be improved by undertaking exercise [38]. Accessibility to exercise facilities [37, 39], neighborhood safety [40], negative perception of exercise outcome [39], and inadequate skill and resource [41] were found to be barriers to physical activity in other international studies but not in the current study, which may be due to psychosocial and lifestyle variation between the countries.

One of the limitations of this study is that it is based on self-reported data and is cross-sectional in nature. Nevertheless, self-report is one of the most feasible and cost-effective methods for collecting data and can provide actionable information despite limitations [42]. Often, self-reported data on adherence behavior are overestimated [42] which may be more prominent in our study since all of our study participants were advised by their physician to be physically active and eat healthier. Furthermore, findings may be limited in generalizability as data was collected from a private diabetic clinic in an urban area. Small sample size is also a limitation. Nevertheless, this study provides preliminary findings on barriers in the Nepalese social context and has great potential to provide information for clinical practice in Nepal.

## 5. Conclusions

Perceived social acceptability, reminder to action, lack of taste and knowledge, and availability of healthy options were important barriers to compliance to a healthy diet. Self-efficacy and social acceptability were significant barriers to compliance to exercise. The supportive role of children and spouses and the opposing role of friends and relatives were important for compliance to both.

This study is first of its kind in Nepal and is important for many reasons. First, patients have the greatest responsibility for adherence [43], and their perceptions and beliefs are critical to their health overall and health behaviors specifically [44]. Therefore, it is important to identify and address the barriers, as perceived by the patients themselves. Secondly, addressing these barriers by targeting populations with relevant messages, even very difficult behaviors can be changed. The findings also suggest that social embeddedness influences compliance among participants. Therefore, the social model of health should be integrated into programs aiming to promote a healthy lifestyle among Nepalese T2DM patients. Since healthy diet and physical activity play an important role in the etiology of most chronic diseases, the barriers identified in this study not only are relevant to diabetes management but could also contribute to the effective prevention of other NCDs.

*What Is Already Known about the Topic?*

- The health benefits of healthy diet and physical activity, in general, and among diabetic patients, are well documented.
- The sociodemographic characteristics associated with adherence to diet and physical activity among Nepalese diabetic patients have been established.

*What Does This Paper Add?*

- The psychosocial and other modifiable risk factors that prevent T2DM patients in Nepal from adhering to diet and exercise advice.
- Sociocultural rather than individual characteristics were significant barriers to diet and exercise compliance among Nepalese T2DM patients.

## Figures and Tables

**Table 1:** Characteristics of study participants.

	Total (*n* = 197)	Diet			Exercise		
		Compliant (*n* = 116)	Noncompliant (*n* = 81)	^a^ *P* value	Compliant (*n* = 107)	Noncompliant (*n* = 90)	^b^ *P* value
*Age, Mean ± SD *	54.7 ± 11.3	55.3 ± 11.4	53.8 ± 11.3	0.340^*∗*^	54.1 ± 11.3	55.3 ± 11.5	0.459^*∗*^
*Sex, n (%)*							
Male	111 (56.3)	67 (60.4)	44 (39.6)	0.632	59 (53.2)	52 (46.8)	0.71
Female	86 (43.7)	49 (57.0)	37 (43.0)		48 (55.8)	38 (44.2)	
*Ethnicity, n (%)*							
Upper caste groups	79 (40.1)	46 (58.2)	33 (41.8)	0.093	47 (59.5)	32 (40.5)	0.103
Janajati	101 (51.3)	64 (63.4)	37 (36.6)		48 (47.5)	53 (52.5)	
Dalit and minorities	17 (8.6)	6 (35.3)	11 (64.7)		12 (70.6)	5 (29.4)	
*Education, n (%)*							
Illiterate	49 (24.9)	31 (63.3)	18 (36.7)	0.511	27 (55.1)	22 (44.9)	0.546
Formal	109 (55.3)	65 (59.6)	44 (40.4)		56 (51.4)	53 (48.6)	
University	39 (19.8)	20 (51.3)	19 (48.7)		24 (61.5)	15 (38.5)	
*Occupation, n (%)*							
Retired	23 (11.7)	17 (73.9)	6 (26.1)	0.222	13 (56.5)	10 (43.5)	0.762
Professional	24 (12.2)	11 (45.8)	13 (54.2)		15 (62.5)	9 (37.5)	
Agricultural	33 (16.8)	17 (51.5)	16 (48.5)		15 (45.5)	18 (54.5)	
Business	55 (27.9)	36 (65.5)	19 (34.5)		31 (56.4)	24 (43.6)	
Home Maker	62 (31.5)	35 (56.5)	27 (43.5)		33 (53.2)	29 (46.8)	
*Marital status, n (%)*							
Married	197 (100.0)	116 (58.9)	81 (41.1)		107 (54.3)	90 (45.7)	
*Family type, n (%)*							
Nuclear	100 (50.8)	58 (58.0)	42 (42.0)	0.798	60 (60.0)	40 (40.0)	0.104
Joint	97 (49.2)	58 (59.8)	39 (40.2)		47 (48.5)	50 (51.5)	
*Family size, mean ± SD*	5.5 ± 2.5	5.4 ± 2.5	5.7 ± 2.4	0.436^*∗*^	5.5 ± 2.3	5.6 ± 2.7	0.840^*∗*^
*Family income ($), mean ± SD*	360.2 ± 212.1	369.6 ± 231.8	346.8 ± 181.0	0.460^*∗*^	387.0 ± 228.4	328.3 ± 187.3	0.053^*∗*^
*Residence, n (%)*							
Urban	170 (86.3)	101 (59.4)	69 (40.6)	0.705	95 (55.9)	75 (44.1)	0.268
Rural	27 (13.7)	15 (55.6)	12 (44.4)		12 (44.4)	15 (55.6)	
*Smoking, n (%)*							
Yes	46 (23.4)	18 (39.1)	28 (60.9)	**0.003**	25 (54.3)	21 (45.7)	0.527
No	128 (65)	86 (67.2)	42 (32.8)		72 (56.3)	56 (43.8)	
Previously	23 (11.7)	12 (52.2)	11 (47.8)		10 (43.5)	13 (56.5)	
*Alcohol intake, n (%)*							
Yes	76 (38.6)	35 (46.1)	41 (53.9)	**0.013**	42 (55.3)	34 (44.7)	0.116
No	100 (50.8)	68 (68.0)	32 (32.0)		58 (58.0)	42 (42.0)	
Previously	21 (10.7)	13 (61.9)	8 (38.1)		7 (33.3)	14 (66.7)	

^*∗*^Independent  *t*-test for mean differences between compliant and noncompliant groups. All others are Chi-square tests. ^a^Group difference between compliant and noncompliant to dietary advice. ^b^Group difference between compliant and noncompliant to exercise advice.

**Table 2:** Barriers to dietary compliance among the respondents.

Determinants of dietary barrier	Yes, *n* (%)	Compliant	Noncompliant Odds ratio (95% CI)	
			Univariate	Multivariate^a^
*Self-efficacy* (participant believed to be capable of dietary compliance)	192 (97.5)	Reference	0.49 (0.09–2.55)	0.30 (0.05–1.64)
*Social acceptability* (believed to have family, community support for dietary compliance)	184 (93.4)	Reference	**0.13 (0.03**–**0.54)**	**0.14 (0.03**–**0.58)**
*Action efficacy* (believed dietary compliance will control blood glucose)	154 (78.2)	Reference	1.08 (0.55–2.14)	1.16 (0.57–2.34)
*Reminder* (believed it is difficult to remember to comply)	48 (24.4)	Reference	**3.15 (1.62**–**6.15)**	**2.77 (1.38**–**5.53)**
*Accessibility of materials* (believed they have access to healthy food options)	28 (14.2)	Reference	0.47 (0.21–1.05)	0.51 (0.22–1.16)
*Perceived severity* (believed diabetes is a serious health problem)	150 (76.1)	Reference	0.74 (0.38–1.42)	0.64 (0.32–1.25)
*Perceived risk* (believed noncompliance to diet will lead to serious diabetes complications)	131 (66.5)	Reference	0.77 (0.42–1.39)	0.73 (0.40–1.36)

^a^Multivariate adjusted for age, gender, education, smoking, and alcohol.

**Table 3:** Participants reason for noncompliance to diet (*n* = 81).

I could not comply with my dietary recommendations because	Agree (%)	Disagree (%)
I don't have knowledge on healthy options for diabetic patients	79.0	21.0
Nobody motivates me to eat healthy diet	35.8	64.2
I don't like to eat my recommended diet	74.1	25.9
I have no knowledge on how to cook/buy diet healthy for diabetic patients	63.0	37.1
I can't eat/buy healthy food easily	41.9	58.0
Healthy diet recommended to me is expensive	56.8	43.2
I am very busy with work and don't have time to buy/cook healthy diet	42.0	58.0
I am very busy with family commitments and don't have time to buy/cook healthy options	28.4	71.6

**Table 4:** Barriers to compliance to exercise among the respondents.

Determinants of Barrier to physical activity	Yes, *n* (%)	Compliant	Noncompliant Odds ratio (95% CI)	
			Univariate	Multivariate^a^
*Self-efficacy* (Participant believed to be capable of compliance to exercise)	178 (90.4)	Reference	**0.10 (0.03**–**0.39)**	**0.09 (0.02**–**0.34)**
*Social acceptability* (Believed to have family, community support for compliance to exercise)	168 (85.3)	Reference	**0.11 (0.04**–**0.32)**	**0.12 (0.04**–**0.34)**
*Action efficacy* (Believed compliance to exercise will control blood glucose)	142 (72.1)	Reference	0.75 (0.40–1.39)	0.77 (0.41–1.47)
*Reminder* (Believed it is difficult to remember to comply)	42 (21.3)	Reference	1.25 (0.63–2.45)	1.19 (0.59–2.38)
*Accessibility of materials* (Believed they have access to resources to exercise)	58 (29.4)	Reference	1.27 (0.69–2.36)	1.25 (0.68–2.32)
*Perceived severity* (Believed diabetes is a serious health problem)	151 (76.6)	Reference	1.41 (0.72–2.74)	1.39 (0.72–2.70)
*Perceived risk* (Believed noncompliance to exercise will lead to serious diabetes complications)	138 (70.1)	Reference	0.82 (0.45–1.51)	0.84 (0.45–1.56)

^a^Multivariate adjusted for age, gender, education, smoking, and alcohol.

**Table 5:** Participants reason for noncompliance to exercise (*n* = 90).

Barriers to being active	*n* (%)
Lack of time	32 (35.6)
Social influence	24 (26.7)
Lack of energy	62 (68.9)
Lack of willpower	69 (76.7)
Fear of injury	33 (36.7)
Lack of skill	31 (34.4)
Lack of resources	33 (36.7)
